# Microalgae: revolutionizing skin repair and enhancement

**DOI:** 10.1016/j.btre.2025.e00911

**Published:** 2025-08-06

**Authors:** Negin Chinjoo, Abooali Golzary

**Affiliations:** a School of Biology, Damghan University, Damghan, Iran; b School of Environment, College of Engineering, University of Tehran, P.O. Box 14155-6135, Tehran, Iran

**Keywords:** Microalgae-derived bioactives, Sustainable skincare solutions, Antioxidants and collagen enhancement, Cosmeceuticals and UV protection, Polysaccharides and antiaging

## Abstract

Microalgae are attracting growing interest in the cosmetics industry due to their rich content of antioxidants, vitamins, and polyunsaturated fatty acids, which offer anti-aging, anti-inflammatory, and photoprotective benefits. Current research primarily focuses on individual species and isolated bioactive compounds. Several challenges persist, including scalability, cost-effective production, low biomass yields, and regulatory constraints. From an environmental perspective, microalgae can be sustainably cultivated on non-arable land, using recycled water and capturing CO₂, thus reducing carbon emissions. Technological advancements, such as photobioreactor systems, have further enhanced resource efficiency and minimized ecological impact. This review explores multiple microalgal species and their combined bioactive potential for innovative cosmetic formulations. By integrating green extraction techniques and circular economy principles, microalgae-based products can meet rising consumer demand for clean beauty while promoting sustainability. With increasing clinical evidence and ongoing technological progress, microalgae hold significant promise for driving eco-friendly innovation in the cosmetics sector.

## Highlights

• Microalgae market in cosmetics projected to surpass USD 1.3 billion by 2030.
• Microalgae bioactives improve skin repair, hydration, elasticity, and UV defense.
• Antioxidants from microalgae reduce oxidative stress and delay skin aging.
• Polysaccharides and fatty acids boost collagen, moisture, and skin resilience.
• Eco-friendly microalgae cosmeceuticals support sustainable beauty innovation.

## 1 Introduction

### 1.1 Definition of microalgae

In recent years, microalgae have attracted considerable attention in the cosmetics and skincare industry due to their diverse range of bioactive compounds and their sustainable cultivation requirements. This review specifically focuses on the cosmetic applications of microalgae, highlighting their potential to replace synthetic and less eco-friendly ingredients traditionally used in skincare products [1,2]. Microalgae, eukaryotic and microscopic, exhibit a remarkable size range from 0.2 µm to over 70 mm. These polyphyletic and photosynthetic organisms, including brown, red, green, and golden algae, prokaryotic cyanobacteria (blue-green algae), diatoms, and dinoflagellates, play a crucial role in environmental sustainability and nutraceutical applications [[3], [4], [5]]. Microalgae demonstrate a remarkable capacity to inhabit many environments, including extensive marine and freshwater ecosystems, as well as complex niches in moist soils and hypersaline settings. They can even flourish in the distinctive microhabitats present on rock surfaces, tree bark, and within specific animal hosts. This remarkable adaptability highlights their ecological versatility and resilience, enabling them to flourish in diverse and often extreme conditions [[6], [7], [8], [9], [10]]. Due to this adaptability, microalgae encompass a vast array of species capable of living in numerous environments, with notable examples listed in Table 1 [10]. Microalgae are foundational to aquatic food webs, converting solar energy into organic matter and supporting a broad range of aquatic life [6].

**Table 1:** Main classes and species of microalgae.

Class	Notable species
*Chlorophyceae (Green Algae)*	*Chlorella vulgaris, Dunaliella salina, Haematococcus pluvialis*
*Bacillariophyceae (Diatoms)*	*Thalassiosira pseudonana, Phaeodactylum tricornutum, Navicula incerta*
*Cyanophyceae (Blue-Green Algae, Cyanobacteria)*	*Spirulina platensis, Anabaena cylindrica, Nostoc commune*
*Rhodophyceae (Red Algae)*	*Porphyridium cruentum, Gracilaria gracilis, Palmaria palmata*
*Phaeophyceae (Brown Algae)*	*Laminaria digitata, Macrocystis pyrifera, Sargassum muticum*
*Chrysophyceae (Golden Algae)*	*Isochrysis galbana, Prymnesium parvum, Ochromonas danica*

As some of Earth's earliest life forms, microalgae have thrived for over 3 billion years, contributing significantly to the planet's biodiversity with an estimated 200,000 to 800,000 species, though only 35,000 are documented [4,11]. These versatile organisms require sunlight, water, and a carbon source to thrive, and they are instrumental in producing approximately 50 % of Earth's oxygen, reducing atmospheric carbon levels and greenhouse gas emissions [10,12]. Their efficiency in photosynthesis and nutrient absorption underscores their commercial and ecological significance [13].

**Fig. 1:**
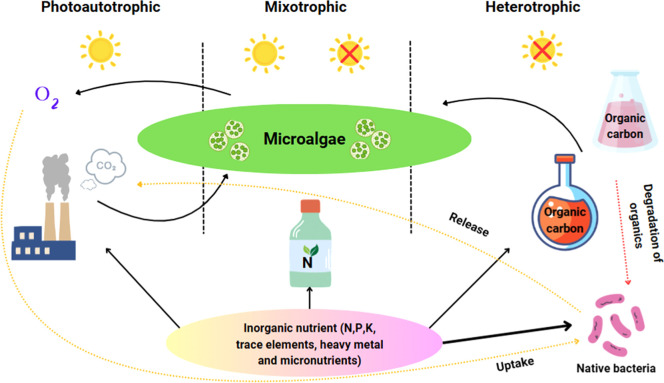
Adaptation strategies of microalgae: autotrophic, mixotrophic, and heterotrophic growth conditions.

Microalgae are increasingly favored in the cosmetics industry over other natural sources due to their rapid growth, high yields of bioactive compounds, low land and water requirements, and potential for sustainable, year-round cultivation. Unlike terrestrial plants, they can be grown in controlled environments without competing with food crops, making them an environmentally sustainable and commercially promising option for future skincare innovations [14,15]. Moreover, microalgae exhibit remarkable physiological and biochemical flexibility, thriving under autotrophic, mixotrophic, or heterotrophic conditions depending on nutrient availability (Fig. 1) [16]. They respond to abiogenic stressors, such as extreme temperatures and nutrient deficiencies, and biogenic challenges, like competition and parasitism, by adjusting cell composition, activating defense mechanisms, and producing bioactive substances, including cytotoxins and antibacterial agents [5].

This adaptability renders them a vital resource for natural, safe, and eco-friendly products, spurring renewed interest in their study and application in recent years [5]. Their comprehensive impact on ecosystems and potential in biotechnology and environmental sustainability make microalgae a subject of significant scientific and commercial interest [13].

### 1.2 Importance of microalgae in various industries

**Fig. 2:**
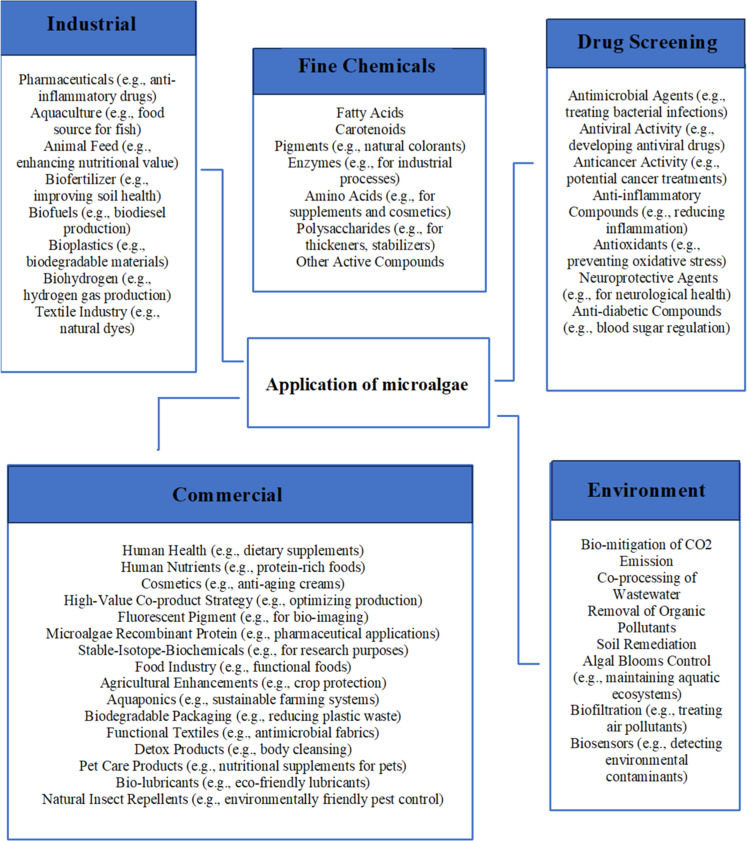
Diverse and innovative industrial applications of microalgae.

Microalgae have been harnessed since the 1950s for the production of valuable bioactive compounds [17]. The increasing need for environmental protection and renewable energy has recently renewed interest in microalgae research [16]. These organisms are pivotal to global ecosystems, contributing to approximately half of the world's annual primary production [7]. Microalgae are incredibly versatile, capable of producing a diverse array of essential compounds utilized across various industries, including food, cosmetics, animal feed, biofuels, nutraceuticals, pharmaceuticals, textiles, and agriculture. They play a crucial role in biofuel production, carbon dioxide reduction, and environmental remediation [18]. These microorganisms effectively treat wastewater and industrial emissions, such as flue gas, by converting carbon dioxide into valuable products, underscoring their importance across multiple fields Fig. 2 [12].

Microalgae possess several advantages over macroalgae and terrestrial plants. They are more efficient in photosynthesis, grow faster, contain higher concentrations of valuable compounds, and require less space or land [7]. However, the production of energy derivatives from microalgae remains economically challenging unless integrated with the production of other valuable compounds. The commercialization of microalgal products faces hurdles such as small market size, high production costs, low biomass and product yield, and stringent regulatory requirements [19,20].

Increasingly, microalgae are employed in the production of recombinant proteins due to their efficiency as bioreactors. Advanced genetic engineering techniques, including CRISPR/Cas9 and other transformation methods, are utilized to enhance the yield of valuable compounds [21,22]. Microalgae produce a range of bioactive compounds with promising health benefits, including antioxidant, antimicrobial, anti-inflammatory, antiviral, and anticancer properties. Recombinant proteins derived from microalgae find applications in pharmaceuticals, nutrition, and the feed industry [23]. Additionally, microalgae are invaluable in isotope biochemical studies, providing insights into metabolic pathways and biochemical processes [24].

Their commercial potential in the food industry is expected to expand. In aquaculture, microalgae are explored as nutraceuticals to enhance the immune responses of marine animals, though challenges in production, concentration, and storage costs remain [23].

### 1.3 Role of microalgae in beauty and skincare

Microalgae species are currently prominent in various cosmetic and skincare products, serving as reliable organic ingredients that enhance their value [25]. These microorganisms possess exceptional biological properties, providing a rich source of compounds such as polysaccharides, lipids, proteins (amino acids), carotenoids, and phycobiliproteins. Extensively studied for their potential in cosmeceuticals, microalgae seamlessly blend the benefits of cosmetics and pharmaceuticals [5]. They are also used as stabilizers, dyes, or thickening agents [25]. Their sustainable harvesting methods and avoidance of animal testing make them highly attractive as premium ingredients in vegan cosmetics. With a global trend towards wellness and natural beauty, microalgae's unique attributes offer a compelling advantage for brands aiming to resonate with today's informed consumers [26]. A significant advantage of microalgae in the cosmetics industry is their ability to endure and recover from harsh environmental conditions, protecting skin cells from damage and reducing harmful free radicals. These characteristics make them excellent alternatives to synthetic products, which can negatively impact the skin. In cosmetics, microalgae can be utilized directly or through their active compounds [20].

The global microalgae market for cosmetics was valued at approximately USD 1.2 billion in 2023 and is projected to grow at a Compound Annual Growth Rate (CAGR) of 8.5 % through 2030, driven by rising consumer demand for sustainable and natural ingredients. This growth is largely attributed to the incorporation of microalgal extracts into various skincare formulations, including anti-aging creams, sunscreens, hydrating serums, and pigmentation treatments. The rich profile of bioactive compounds such as antioxidants, polyunsaturated fatty acids, peptides, and mycosporine-like amino acids (MAAs) makes microalgae highly effective in addressing diverse skin concerns [27]. Leading cosmetic companies such as L’Oréal, Unilever, La Prairie, Nuxe, Algenist, and Pentapharm have already introduced microalgae-based products into their portfolios, underscoring the commercial viability and growing market acceptance of these ingredients . These products range from skin brightening solutions and firming creams to eye contour treatments and UV-protective formulations . In parallel, scientific studies continue to validate the efficacy of microalgae in skincare, with clinical trials reporting improvements in hydration, wrinkle reduction, elasticity, sebum regulation, and photoprotection. Their ability to produce valuable metabolites like astaxanthin, lutein, phycocyanin, and MAAs under controlled conditions further supports their potential for scalable and eco-friendly cosmetic development [28,29].

The cosmetic industry extensively uses microalgae and their bioactive components, which provide various benefits such as antioxidant properties, free-radical scavenging, stress protection, anti-aging solutions [30], sunscreen capabilities, and pigmentation for makeup. They also contribute to immune system enhancement, odor neutralization, product balancing, and detoxification. Microalgae-derived active ingredients offer numerous skin benefits, including reducing inflammation, preventing blemishes, repairing skin, retaining moisture, and improving seborrhea. They also expedite the healing process. Table 2 provides examples of microalgae species used in cosmetics and their potential applications in cosmeceuticals [30].

**Table 2:** Overview of microalgal-based cosmetic products and their skincare benefits.

Microalgae species	Manufacturer	Product	Function and benefits	Ref
*Scenedesmus obliquus*	Unilever, L'Oréal (UK/Netherlands, France)	Skin brightening products	Enhances skin tone, diminishes pigmentation, and promotes a luminous complexion.	https://www.unilever.com/ https://www.loreal.com/
*Chlamydomonas nivalis*	La Prairie (Switzerland)	Cellular Swiss ice crystal dry oil	Anti-aging.	https://www.laprairieswitze rland.com
*Haematococcus pluvialis*	Patyka (France)	Patyka Face Sun Cream SPF50	UVA/B protection, moisturizer	^https://patyka.com/^
*Spirulina*	Optimum Derma Aciditate (Lithuania)	Skin whitening facial mask, Algae mask	Moisturizer, boosts immunity, refines complexion, and diminishes wrinkles	^https://optimumderma^
*Spirulina*	PuroBIO Cosmetics (Italy)	KELLY powder mask	Peel-off mask designed specifically for dry skin.	^https://purobiocosmetics.it/^
*Spirulina*	Santè Naturels (Italy)	*Spirulina* Santè Metode face line	Anti-aging elixir, harmonizing cleansing milk, rejuvenating tonic, antioxidant powerhouse	^https://www.santenaturels.it/^
*Spirulina*	REN Skincare (UK)	Mattifying Clay Purifying Mask	Targets blemishes, reduces the visibility of pores, eliminates excess sebum, and combats congestion, hydration	^https://www.renskincare.com/^
*Spirulina maxima*	Ocean Pharma (Germany)	Skinner	Skin renewal and inherent defense	^https://www.ocean-pharma.de/^
*Spirulina*	Algotherm (France)	ocean cleanse 3-in-1 Active Shield Serum	Helps combat signs of premature aging (wrinkles, fatigue, dull complexion, spots)	^https://www.algotherm.com/^
*Green Micro-Algae & Brown Algae*	Algotherm (France)	ocean cleanse 2-in-1 [Purifying] Scrub	Exfoliation, skin regulation, stimulate cell renewal, improving texture and reducing signs of aging, regulate sebum production, reducing shine and preventing blemishes	^https://www.algotherm.com/^
*Chlorella vulgaris*	BASF SE, DIC Corporation (Germany, Japan)	Moisturizers, anti-aging creams	Skin rejuvenation, hydration, and reducing the appearance of aging	^http://www.basf.com/global/en^ ^http://www.dic-global.com/en/^
*Chlorella vulgaris*	Phytomer (France)	Phytomer	Enhances the skin's innate defenses and modulates inflammatory responses	^https://www.phytomer.fr/en/^
*Chlorella vulgaris*	Nuxe (France)	Merveillance Lift Firming Activating Oil-Serum	Firming and strengthening the skin, containing microalgae oil, improving skin elasticity	^https://us.nuxe.com/^
*Chlorella vulgaris*	Nuxe (France)	Merveillance Lift Firming Powdery Cream	Reducing wrinkles, deep hydration, firming the skin	^https://us.nuxe.com/^
*Chlorella vulgaris*	Nuxe (France)	Merveillance Lift Eye Contour Cream	Reducing puffiness and dark circles, diminishing fine lines around the eyes	^https://us.nuxe.com/^
*Chlorella vulgaris*	Nuxe (France)	Merveillance Lift Concentrated Night Cream	Nourishing and repairing the skin overnight, containing antioxidants, reducing wrinkles	^https://us.nuxe.com/^
*Chlorella*	Algenist (United States)	Green Microalgae Retinol + Regenerating Serum	Diminishes the appearance of deep lines and wrinkles, improves skin texture and tone, promotes accelerated skin cell turnover	^https://www.algenist.com/^
*Chlorella*	Algenist (United States)	POWER Recharging Night Pressed Serum	Recharges fatigued skin, minimizes fine lines, refines texture overnight	^https://www.algenist.com/^
*Saccharum Officinarum*	Algenist (United States)	Concentrated Reconstructing Serum	Anti-aging and diminishes the appearance of deep lines and wrinkles	^https://www.algenist.com/^
*Nannochloropsis oculata*	Pentapharm Ltd (Switzerland)	Pepha-Tight	Enhances the skin's tightening efficacy	^https://www.pentapharm.com/^
*Dunaliella salina*	Pentapharm Ltd (Switzerland)	Pepta-Ctive	Promotes epidermal cell proliferation and enhances the skin's energy metabolism	^https://www.pentapharm.com/^
*Dunaliella salina*	Algenist (United States)	Color-Correcting Eye Brightener	Concealer that minimizes uneven skin tone around the eyes and effectively conceals dark circles	^http://www.algenist.com/^
*Dunaliella salina*	Cargill, Incorporated (United States)	Sunscreens, anti-cellulite products	UV protection, enhances skin elasticity and smoothness	^https://www.cargill.com/^
*Dunaliella salina*	Ahava (Israeli)	*Dunaliella* Algae Peel-Off Mask	Lifts away fatigue and dullness, helps against blackheads and clogged pores, refines and smoothes the skin’s surface	^https://global.ahava.com/^
*Anacystis nidulans*	Estee Lauder (USA) Solazyme (France)	Photosomes Algenist	Enhances the skin's immune response and provides effective sun protection	^https://www.esteelauder.com/^ ^https://www.solazyme.com/^
*Spirulina platensis*	Exsymol S.A.M (Monaco) Sanatur GmbH (Germany)	Protulines	Prevents premature skin aging and the formation of wrinkles	^https://www.exsymol.com/^ ^https://www.sanatur.de/^
*Spirulina platensis*	Bluetec Naturals Co., Ltd (South Korea)	Nutritional supplements, skin treatments	Moisturizer, alleviates inflammation, and delivers vital amino acids and minerals for holistic skin health	^https://www.bluetecnaturals.com/^
*Spirulina platensis*	Iraya Skincare (Sweden)	Iraya Body Lotion	Hydration and moisturizer	^https://irayeskincare.com/^
*Nannochloropsis oculata*	Evonik Industries (Germany)	Anti-wrinkle creams	boosts skin elasticity and diminishes fine lines and wrinkles	^https://www.evonik.com/^
*Porphyra umbilicalis*	Jenelt (South Korea)	Sunscreen SPF 30	UV protection	^http://www.jenelt.com/^
*Tetraselmis suecica*	Croda International (United Kingdom)	Hydrating serums	Hydration, moisturizer and strengthens the skin barrier	^https://www.croda.com/en-gb^
*Phaeodactylum Tricornutum*	Be Mused Korea (Korea)	Oloa Microalgae Duo Set	Hydration and moisturizer, firmer skin, bouncy skin, youthful appearance, regenerative microalgae benefits, revitalizing dull skin	^https://bemusedkorea.com/^

## 2 Active compounds in microalgae

Microalgae are powerhouses of bioactive compounds. They are rich in antioxidants, vitamins, minerals, peptides and amino acids, essential fatty acids (EFAs), and polysaccharides, all of which contribute significantly to skin health and cosmetic applications Table 3 [31].

**Table 3:** Beneficial compounds found in microalgae.

Beneficial compound	Examples	Ref
Antioxidants	Pigments like chlorophyll, astaxanthin, zeaxanthin, β-carotene, phycocyanin, lutein, phenolic compounds, phycoerythrin, fucoxanthin, canthaxanthin and enzymes like superoxide dismutase (SOD), catalase (CAT), glutathione (GSH), and glutathione peroxidase (GPX)	[[28], [29], [30], [31], [32], [33], [34]]
Vitamins	Pro-vitamin A, vitamin C, B1, B2, B3, B9, B12, D, E, K,	[35,37]
Minerals	Potassium, iron, magnesium, calcium, iodine, niacin, nicotinate, biotin, zinc, selenium, phosphorus, sodium, copper, sulfur	[28,39]
Essential Fatty Acids	Omega-3 (EPA (Eicosapentaenoic Acid)), DHA(Docosahexaenoic Acid)), omega-6 (linoleic acid)	[47]
Polysaccharides	Galactose, xylose, glucose, rhamnose, fucose, arabinose, mannose, ortho-methyl sugar, exopolysaccharides, alginate, carrageenan, chitin, chitosan	[55,56,[60], [61], [62], [63], [64], [65], [66]]
Peptides and Amino Acids	Phycocyanin, allophycocyanin, lectins, alanine, serine, proline, histidine, taurine, tyrosine, tryptophan	[[78], [79], [80]]

### 2.1 Antioxidants

In living organisms, a sophisticated antioxidant defense system, comprising various antioxidants and enzymes, maintains the delicate balance between the generation and breakdown of reactive oxygen species (ROS) [32]. Microalgae, known for producing antioxidants in response to abiotic stress, effectively mitigate oxidative damage [33]. Microalgae contain compounds such as pigments, which are used as antioxidants in the cosmetic industry. These high-value pigments are used in cosmetics like moisturizers and sunscreens, helping prevent and treat conditions from photoaging to skin cancer, while also acting as natural colorants [33,34].

Photosynthetic pigments, including carotenoids, play an essential role. For example, β-carotenes from *Dunaliella salina* and astaxanthin from *Haematococcus pluvialis* (which is significantly more potent than other carotenoids like β-carotenes and zeaxanthin), as well as phycoerythrobilins from *Spirulina* and *Porphyridium*, are noted for their antioxidative properties. Recent studies suggest that astaxanthin and β-carotenes exhibit a synergistic effect, enhancing antioxidant capacity and improving protection against oxidative stress, environmental damage, and premature aging. This improved oxidative defense mechanism contributes to better skin health and prolonged elasticity, particularly in skincare formulations designed for anti-aging treatments [35].

Notably, phycobiliproteins (e.g., phycoerythrin) show pH-dependent stability, making them suitable for acidic formulations like serums and toners. Phycobiliproteins from *Porphyridium perineum* are frequently used as coloring agents in cosmetic products, retaining their color stability even within a pH range of 4 to 5 and under light exposure [36]. Chlorophyll and its derivatives, naturally synthesized by various microalgal species, are well-regarded for their antioxidant properties. Fucoxanthin and its related compounds, such as auroxanthin, demonstrate exceptional radical-scavenging abilities. Notably, fucoxanthin exhibits even greater antioxidant activity than β-carotene. However, formulation research suggests that excessive β-carotene concentrations may compromise fucoxanthin’s bioavailability, highlighting the need for an optimal balance in cosmetic applications [37].

Pigments in microalgae, such as phenolic and alkaloid compounds, exhibit antioxidant properties by donating hydrogen atoms to unstable free radicals. The antioxidant capacity of microalgae is influenced not only by the concentration of these phenolic compounds but also by their specific types within the biomass [38]. Additionally, microalgae are recognized as a primary natural source of lutein, often producing higher levels of this compound than plants [39]. Microalgae-derived antioxidants can stimulate the activity of endogenous antioxidant enzymes like catalase (CAT), superoxide dismutase (SOD), glutathione (GSH), and glutathione peroxidase (GPX). These enzymes collaborate to neutralize ROS and maintain redox balance in cells. Superoxide radicals are converted into hydrogen peroxide by SOD, which GPX and CAT then break down into oxygen and water, thus protecting cells from damage. Working synergistically, these enzymes reduce inflammation, enhance skin vitality, and combat signs of aging. Consequently, microalgae-derived antioxidants are valuable in skincare products for maintaining skin elasticity and reducing wrinkles [[40], [41], [42]].

### 2.2 Vitamins and minerals

Vitamins are indispensable for maintaining health, acting as essential components in enzyme functions during metabolic processes, and exhibiting potent antioxidant properties [43]. Microalgae, which are nutrient-dense microorganisms, often contain higher levels of essential vitamins compared to land plants. These nutrients include precursors to vitamin A, vitamin C, and a variety of B vitamins such as thiamine (B1), folic acid (B9), riboflavin (B2), niacin (B3), cobalamin (B12), and α-tocopherol (E), as well as vitamins D and K. The wide range of vitamins present in microalgae makes them a valuable resource for producing natural vitamins suitable for human consumption [44,45]. Emerging studies highlight the potential for synergistic interactions between vitamins in microalgae, improving both their effectiveness and bioavailability. For instance, the combination of β-carotene-rich *Dunaliella* and B-vitamin-rich *Chlorella* extracts may enhance nutrient stability and absorption, particularly when paired with mineral chelators that facilitate penetration into the skin in cosmetic formulations. Additionally, vitamins can work synergistically with α- and β-carotene, amplifying their antioxidant effects and improving nutrient utilization in biological systems. Environmental variables such as temperature, nutrient availability, light intensity, and salinity significantly influence vitamin synthesis in microalgae. Additionally, both the growth stage and genotype of each species play a crucial role in determining the quality and concentration of these micronutrients [43,44].

Microalgae-derived vitamins contribute substantially to skin health by reinforcing collagen production, preserving elasticity, and supporting cell repair mechanisms. Synergistic effects between vitamins A, C, and E are particularly beneficial for skincare applications—vitamin C regenerates oxidized vitamin E, creating a continuous antioxidant cycle that strengthens skin protection against oxidative damage. This extended defense mechanism enhances the skin’s resilience and promotes a youthful appearance. Furthermore, minerals such as zinc and selenium aid in collagen synthesis and UV protection, reducing premature aging and shielding against environmental stressors [46].

Certain microalgae species, including *Arthrospira, Chlorella*, and *Scenedesmus*, exhibit higher levels of vitamins A, B1, B2, E, and niacin compared to terrestrial plants, making them valuable sources for natural supplementation. *Dunaliella tertiolecta* and *Tetraselmis suecica*, widely used in aquaculture, provide abundant concentrations of vitamins B12, B2, and E. An endogenous synergy exists between B-complex vitamins and vitamin E, where vitamin E prevents thiamine oxidation during storage, preserving its bioactivity in formulations [33,34].

Beyond vitamins, microalgae are abundant sources of essential minerals such as potassium, iron, magnesium, calcium, iodine, biotin, and nicotinate, etc. Their mineral content constitutes approximately 2.2 to 4.8 % of dry biomass, including elements such as zinc, sulfur, copper, and phosphorus [33,[47], [48], [49]].

By leveraging their high nutritional density, environmental resilience, and bioactive properties, microalgae provide a sustainable platform for enhancing health and skincare formulations. Their ability to efficiently produce essential nutrients positions them as promising candidates for future innovations in cosmetics, pharmaceuticals, and nutritional sciences [[47], [48], [49]].

### 2.3 Essential fatty acids

Microalgae are abundant sources of EFAs, particularly omega-3 long-chain polyunsaturated fatty acids such as α-linolenic acid, and omega-6 fatty acids like linoleic acid. These EFAs are vital for maintaining the structural integrity and barrier function of the skin, which helps prevent moisture loss and shields against environmental irritants [50,51]. Since humans cannot synthesize certain EFAs, obtaining them from external sources is crucial. Omega-3 fatty acids, such as eicosapentaenoic acid (EPA) and docosahexaenoic acid, have notable anti-inflammatory properties. They inhibit the production of inflammatory cytokines and eicosanoids, alleviating conditions like psoriasis, acne, and eczema. This results in clearer, calmer skin and overall better skin health by keeping the skin hydrated and reducing irritation [52].

EFAs play an essential role in maintaining skin hydration by reinforcing the lipid barrier, thereby preventing transepidermal water loss (TEWL). This process keeps the skin soft, smooth, and supple. Adequate levels of EFAs are necessary to keep the skin moisturized, reducing dryness and flakiness. Furthermore, EFAs support the synthesis of new skin cells and collagen, promoting faster wound repair and reducing the risk of infection and scarring [48]. By providing the necessary building blocks for cell membranes, EFAs help maintain the skin's structural integrity and resilience against environmental factors. This reinforcement of the lipid barrier is especially important for individuals with sensitive or dry skin, as it helps to lock in moisture and protect the skin from external irritants [53,54]. Emerging evidence suggests that combining microalgae-derived EFAs with antioxidant pigments like astaxanthin may provide dual protection against both UV-induced damage and TEWL [55].

Omega-6 fatty acids are crucial for the creation of ceramides, lipids that are essential for maintaining the skin's barrier function. These ceramides play a critical role in locking in moisture and protecting the skin from environmental factors, ensuring that the skin remains hydrated and healthy. Without adequate levels of omega-6 fatty acids, the skin can become dry and prone to irritation. On the other hand, omega-3 fatty acids protect against UV-induced skin damage by minimizing oxidative stress and inflammation, both of which are significant contributors to photoaging and skin damage. By reducing the harmful effects of UV radiation, omega-3 fatty acids help to maintain a youthful appearance and prevent premature aging. Regular intake of omega-3 fatty acids can also help alleviate symptoms of inflammatory skin conditions, offering relief to those suffering from chronic skin issues. The combination of omega-3 and omega-6 fatty acids in skincare formulations can create a synergistic effect, enhancing overall skin health, hydration, and protection against environmental damage [56,57]. Clinical observations show that maintaining a specific omega-3 to omega-6 ratio (approximately 1:1 to 1:4) maximizes this synergy while preventing potential inflammatory responses from omega-6 dominance [58].

### 2.4 Polysaccharides

Polysaccharides are indeed polymers, large hydrophilic carbohydrate molecules that are crucial for the structural support and protection of living organisms [43]. In microalgae, the makeup of these polysaccharides, such as exopolysaccharides (EPS), is influenced by factors like temperature, light intensity, metal ions, and nutrient availability. EPS are copiously secreted into the extracellular environment, acting as a shield against fluctuating environmental conditions and potential threats. These polysaccharides can be grouped into two categories based on their composition: homopolysaccharides, which consist of one type of monosaccharide, and heteropolysaccharides, which comprise multiple types of monosaccharides [59,60].

Key components of microalgal polysaccharides include glucose, galactose, and xylose, linked by glycosidic bonds. Other sugars such as mannose, fucose, rhamnose, arabinose, ortho-methyl sugars, galacturonic acid, and glucuronic acid can also be found. While glucose is predominant, fructose is typically absent in microalgal polysaccharides but is common in those synthesized by cyanobacteria [[61], [62], [63]].

Polysaccharides derived from microalgae are beneficial for skin health due to their ability to enhance skin elasticity and reduce fine lines and wrinkles. They stimulate the production of essential extracellular matrix (ECM) components like collagen and elastin, which provide structural support to the skin, leading to a firmer and more youthful appearance. Moreover, EPS from microalgae is excellent at retaining moisture, drawing it from the environment to maintain skin hydration. This makes them valuable ingredients in skincare products such as moisturizers and serums, ensuring the skin remains smooth and supple. A well-hydrated skin barrier also helps preserve skin flexibility and overall condition [[64], [65], [66]].

Additionally, polysaccharides like alginate and carrageenan, sourced from microalgae, enhance wound healing by promoting cell proliferation and collagen synthesis, essential for tissue repair. When formulated together, these polysaccharides demonstrate a remarkable synergy where alginate provides structural support while carrageenan enhances growth factor retention at the wound site, potentially accelerating healing processes [67].

Studies reveal that *Chlorella pyrenoidosa* produces heteropolysaccharides composed of various sugars, including galactose, fucose, rhamnose, xylose, mannose, glucuronic acid, glucose, and glucosamine [68]. Similarly, *Spirulina platensis* primarily contains glucose and rhamnose, along with other sugars such as mannose, fucose, xylose, glucosamine, and glucuronic acid. Further research indicates that glucose is the dominant monosaccharide in the extracellular polymeric substances of *Spirulina* [69].

EPS from microalgae like *Porphyridium cruentum* and *Glossomastix sp*. are known to boost collagen production in human skin cells by inhibiting matrix metalloproteinase-1 (MMP-1), an enzyme that degrades collagen [70]. Alginate, derived from brown algae (Phaeophyta), is extensively used in pharmaceuticals and biotechnology for its gelling and fiber-forming properties [71]. Ulvan, extracted from green algae (Chlorophyta), is noted for its anti-inflammatory and antioxidant properties, showing potential in biomedical applications [72]. Additionally, some microalgae produce chitin and its derivative chitosan, which are utilized in various biomedical fields [73]. Combining different polysaccharides like alginate and ulvan can create synergistic effects, enhancing their overall effectiveness in wound healing, moisturizing, and anti-aging applications. A promising direction for future cosmetic formulations involves engineered polysaccharide blends derived from multiple microalgae species. Combinations like *Porphyridium* EPS and *Chlorella*-derived glucosamine could pave the way for "smart" hydrogels that dynamically adapt to skin pH and moisture levels, enhancing hydration and barrier function. Exploring these bio-inspired materials may lead to innovative skincare solutions tailored to individual skin needs.

### 2.5 Peptides and amino acids

Microalgae present a sustainable and innovative alternative source of proteins, potentially replacing conventional options. Extracted protein products from microalgae include whole-cell, isolated, hydrolyzed, concentrated proteins, and peptides with bioactive properties [74]. Amino acids and their derivatives, components of the skin's natural moisturizing factor (NMF), play a crucial role in cosmetic formulations by enhancing skin hydration [75]. They are also known to stimulate collagen synthesis in the skin, thereby improving skin health and resilience [76].

Due to the challenges posed by the cell wall in digesting whole-cell proteins from microalgae, peptides produced through enzymatic hydrolysis have gained popularity among scientists [77]. These bioactive compounds exhibit antioxidant, anti-aging, anti-inflammatory, and moisturizing effects. For example, proteins and peptides derived from microalgae effectively scavenge free radicals, thereby reducing oxidative stress and slowing premature skin aging [78]. They also stimulate collagen synthesis, which enhances skin elasticity and firmness, and reduces wrinkles [64]. Additionally, these compounds possess anti-inflammatory properties, making them effective in treating conditions like acne and rosacea [79]. They enhance the skin's capacity to retain moisture, keeping it hydrated and smooth, and promote faster healing of wounds and scars by aiding cell regeneration and repair processes [80]. Microalgae-derived peptides, used in modern cosmetics, particularly skincare products, are formulated as blends of multiple protein fragments produced by breaking down larger proteins through hydrolysis using either chemical or enzymatic techniques [81]. Strategic combination of peptides from different microalgae species (e.g., *Spirulina*'s phycocyanin peptides with *Chlorella*'s CGF) appears to enhance collagen production more effectively than single-source peptides, possibly through complementary activation of different growth factor pathways.

In the skincare world, *Spirulina* and *Chlorella* stand out due to their potent proteins and peptides. *Spirulina* is known for its richness in phycocyanin and allophycocyanin, both of which exhibit strong antioxidant and anti-inflammatory effects [82,83]. It is also abundant in proteins, which can comprise up to 70 % of its dry weight. The enzymatically digested protein hydrolysates of *Spirulina* enhance skin hydration and help manage osmotic stress by regulating water balance genes in skin cells [84]. *Chlorella* contains the *Chlorella* Growth Factor (CGF), which promotes cell regeneration and improves skin elasticity [85]. *Chlorella*-derived peptides are particularly effective at reducing harmful proteins caused by UVB exposure, restoring pro-collagen levels in skin cells, and providing protection against UVC-induced cell damage [86,87].

Apart from *Chlorella* and *Spirulina*, other microalgae species also contribute valuable proteins and peptides used in skincare products. *Haematococcus pluvialis*, for example, produces astaxanthin-binding proteins that stabilize astaxanthin, a potent antioxidant that protects the skin from oxidative stress and UV damage [88].

Many microalgae also synthesize chlorophyll-binding proteins with antioxidant properties that help detoxify the skin [89]. The beneficial effects of these proteins are largely attributed to their amino acid composition, including histidine, serine, taurine, and proline—amino acids known for their hydrophilic or hygroscopic properties that contribute to skin hydration. Others, such as alanine, tyrosine, and tryptophan, play structural or metabolic roles but do not directly participate in skin moisturization [90,91]. Hydrolyzed proteins from these microalgae enhance moisture retention and improve skin texture, while small peptides stimulate collagen production and aid in wound healing [92]. Additionally, lectins, carbohydrate-binding proteins found in various microalgae, offer anti-inflammatory benefits that reduce skin inflammation and promote overall skin health [93].

It seems that integrating diverse peptides and amino acids from microalgae—such as phycocyanin, CGF, and astaxanthin-binding proteins—unlocks powerful synergistic effects. This combination boosts their collective antioxidant, anti-inflammatory, and moisturizing capabilities, providing comprehensive skincare solutions that tackle a variety of skin issues at once.

## 3 Mechanisms of microalgae's effects on skin

### 3.1 Anti-inflammatory properties

Inflammation serves as a natural defense mechanism of the immune system, aiming to restore balance when foreign pathogens disrupt cellular equilibrium. Striving to restore balance when foreign pathogens affect cellular health. This response plays a vital role in various skin ailments such as psoriasis, dermatitis, and acne. Triggered immune responses can spark inflammatory processes, causing tissue damage and worsening these skin conditions [94,95].

Microalgae support skin resilience by influencing molecular pathways involved in inflammation regulation. Their effects are primarily linked to signaling mechanisms that modulate immune responses, rather than relying solely on their antioxidant components. By targeting key cellular pathways, they prevent prolonged inflammation, stabilize skin barrier function, and enhance recovery [[96], [97], [98], [99]]. One major trigger for inflammation is oxidative stress, which damages cellular structures and activates pro-inflammatory messengers. ROS not only cause direct tissue harm but also serve as signaling molecules, amplifying inflammatory responses via distinct molecular pathways [97].

**Fig. 3:**
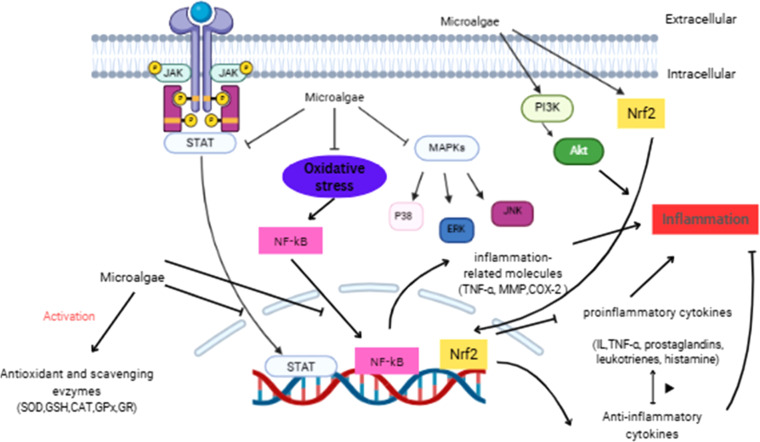
Mechanisms by which microalgae species mitigate skin inflammation. This figure illustrates the involvement of various signaling pathways, antioxidants, cytokines, and chemokines in the anti-inflammatory effects exerted by microalgae extracts. It highlights how these pathways and molecules interact to reduce inflammation, showcasing the multifaceted role of microalgae in modulating inflammatory responses.

Compounds from microalgae reduce skin inflammation through various mechanisms. They can adjust enzyme activities, inhibit nitric oxide synthase (NOS), and regulate cellular processes. Additionally, they target critical signaling pathways like MAPK, NF-κB, PI3K/AKT, and Nrf2. These compounds also reduce free radical production and suppress the expression of pro-inflammatory cytokines, effectively calming inflammation and promoting healthier skin (Fig. 3) [100,101]. The NF-κB signaling cascade plays a central role in inflammatory responses by controlling gene expression related to cytokine production and immune activation. When activated, NF-κB translocates to the nucleus, initiating inflammatory gene transcription. This leads to heightened immune cell activity and prolonged inflammation, exacerbating skin disorders such as eczema and psoriasis [102]. Bioactive compounds derived from microalgae have been shown to modulate NF-κB signaling, thereby contributing to the restoration of cellular homeostasis. Specifically, microalgal polysaccharides inhibit the activation of NF-κB by preventing the phosphorylation of key regulatory proteins, such as IKK-α (IκB kinase alpha) and IκB-α (inhibitor of kappa B alpha), which are essential for initiating the NF-κB pathway. Furthermore, compounds like fucoidans downregulate the expression of NF-κB target genes, including inducible nitric oxide synthase (iNOS) and cyclooxygenase-2 (COX-2), thus attenuating inflammation at the molecular level [[103], [104], [105]].

The MAPK (Mitogen-Activated Protein Kinase) pathway regulates cellular responses to stress, environmental damage, and inflammation. It consists of proteins such as p38 MAPK, ERK, and JNK, which transmit external signals that influence skin cell survival, immune activity, and inflammatory cytokine production [106]. Under oxidative stress, MAPK signaling accelerates inflammation, worsening skin irritation, and impairing collagen stability. Microalgae-derived compounds modulate MAPK activity, reducing stress-related inflammation and protecting skin from environmental triggers. Their effects also extend to JAK/STAT signaling, a pathway influencing immune response intensity and inflammatory cell recruitment [107,108]. The Nrf2 pathway is essential for maintaining cellular health, particularly in defending against oxidative stress-induced inflammation. Upon activation, Nrf2 enhances the production of antioxidant enzymes, which neutralize ROS and prevent inflammatory escalation. Microalgae activate this pathway by stimulating Nrf2-dependent gene expression, reinforcing the skin’s ability to recover from environmental damage. For example, astaxanthin from *Haematococcus pluvialis* has been shown to increase Nrf2 activity, improving cellular resilience and lowering inflammation caused by oxidative stress [[109], [110], [111]].

The PI3K/AKT pathway plays a crucial role in cell growth, repair, and immune regulation, contributing to skin renewal and inflammatory control. Microalgae-derived compounds influence this pathway by supporting epidermal regeneration, reducing cell death caused by oxidative damage, and strengthening the skin’s ability to withstand inflammatory stressors [100].

Inflammation is closely tied to cytokine production, involving mediators like prostaglandins, leukotrienes, TNF-α, and interleukin-1 (IL-1). These molecules amplify immune responses and contribute to chronic inflammation if left unchecked. Additionally, lipid peroxidation, a process in which ROS degrade cell membrane lipids, further intensifies inflammation by generating oxidative byproducts that disrupt cellular function and prolong immune activation. Research highlights that microalgae reduce inflammatory cytokine secretion, preventing excessive immune responses while limiting lipid peroxidation, thus preserving skin integrity. In particular, *Spirulina platensis* produces phycocyanin, which not only lowers the expression of IL-1, IL-6, and TNF-α but also counteracts oxidative damage to membrane lipids, helping alleviate inflammatory conditions such as rosacea and eczema [112,113].

### 3.2 Collagen production enhancement

As skin ages, changes in the dermal ECM lead to the breakdown of key structural elements, including collagen, elastin, and hyaluronic acid. This deterioration results in reduced elasticity, altered enzyme activity, and increased susceptibility to environmental damage [114]. Since collagen accounts for the majority of ECM dry weight, preserving its levels is essential for skin strength, resilience, and overall health [[115], [116], [117]]. Collagen exists in several forms, with fibrillar collagen making up 90 % of total human collagen, primarily located in the ECM. Within this category, type I collagen is the most prevalent, representing 85 % of the total collagen and serving as the foundation of skin connective tissue, ensuring elasticity and durability [118,119]. Type III collagen, another significant component, provides additional support for skin structure, while types IV and VII contribute to basement membrane stabilization [70].

Instead of simply acting as a source of bioactive compounds, microalgae interact with key molecular mechanisms that regulate collagen synthesis and degradation. Their effects extend beyond general antioxidant properties to direct modulation of fibroblast activity and ECM integrity [120,121]. Aging leads to increased activity of collagen-degrading enzymes like MMPs, reducing collagen density and weakening the dermal structure [114]. Microalgae species have been shown to counteract this process by regulating MMP expression, thus preserving skin texture and firmness [122]. For example, *Chlorella* microalgae actively stimulate collagen production, ensuring continuous renewal of the ECM [123]. Additionally, EPS from *Synechococcus sp*., *Glossomastix sp*., *Diacronema sp*., *Pavlova sp*., *Chrysotila dentata, Phaeodactylum tricornutum*, and *Porphyridium cruentum* exhibit the ability to inhibit MMP-1, preventing excessive collagen breakdown while encouraging fibroblast activation [70]. One of the major contributors to collagen deterioration is oxidative stress, which accelerates ECM degradation by promoting lipid peroxidation and increasing inflammatory responses. ROS-induced damage alters the structural integrity of collagen fibers, diminishing skin elasticity over time [124]. Studies show that specific microalgae compounds regulate MMP-1 expression at both transcriptional and protein synthesis levels, ensuring the longevity of collagen networks within the dermis. Research on *Thalassiosira sp*., *Monodus sp*., *Chaetoceros sp*., and *Chlorococcum sp*. supports their potential as ingredients in skincare formulations aimed at reinforcing collagen stability [125].

Additionally, mycosporine-2-glycine (M2G), isolated from microalgae, inhibits collagen-degrading enzymes by chelating calcium ions, preventing excessive protein cross-linking due to glycation. This mechanism helps retain skin radiance and elasticity, making M2G a promising candidate for anti-aging applications [126,127]. Beyond enzyme regulation, microalgae contribute to skin barrier enhancement, protecting against environmental factors such as UV radiation and pollutants. A robust skin barrier retains moisture, preventing dehydration and reinforcing collagen fibers against external damage [128].

Some microalgae-derived peptides also influence collagen renewal by altering ECM-related signaling pathways. Studies indicate that *Chlorella*-derived peptides suppress UVB-induced MMP-1 expression, effectively reducing collagen degradation. This mechanism involves the downregulation of CYR61, a signaling protein associated with ECM remodeling, along with AP-1 and MCP-1, both of which contribute to collagen degradation following UV exposure [129]. By modulating enzymatic activity, stabilizing fibroblast function, and reinforcing skin’s defense systems, microalgae play a critical role in maintaining long-term skin elasticity and firmness. Their ability to support collagen synthesis, regulate degradation pathways, and protect against external damage establishes them as valuable resources for skincare and dermatological applications [130].

### 3.3 UV protection

**Fig. 4:**
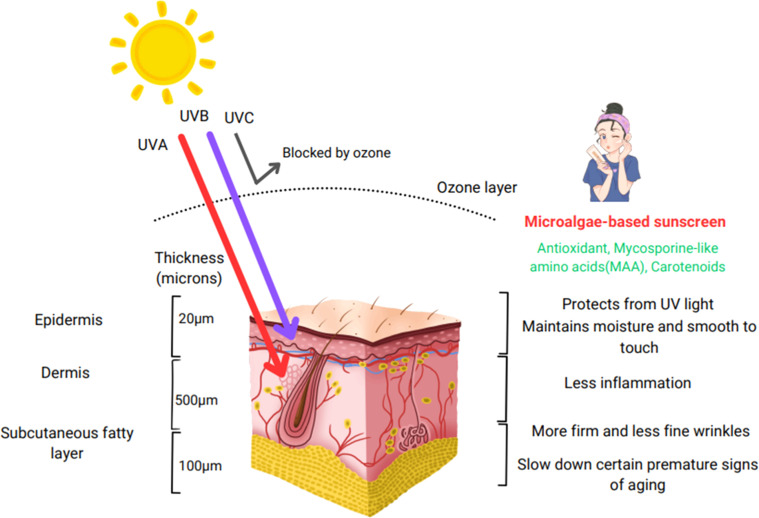
Impact of UV radiation on skin and the role of microalgae-based sunscreen in enhancing skin health.

UV radiation is a major environmental stressor that induces oxidative damage, primarily through the generation of ROS. While acute exposure, such as sunburn, results in immediate effects, long-term exposure leads to premature aging and increases the risk of skin cancers like melanoma [131]. The biological impact of UV-A (320–400 nm) and UV-B (280–320 nm) radiation differs in severity. UV-A penetrates deeper skin layers, contributing to pigmentation changes, dehydration, and wrinkle formation, while also triggering ROS production, which can damage DNA. In contrast, UV-B is highly toxic, affecting upper skin layers, where it causes genetic mutations, abnormal cell growth, and immune suppression by interfering with signal transduction and gene expression (Fig. 4) [132].

Microalgae have evolved multiple protective strategies to counteract UV radiation. They activate enzymes for DNA repair, produce antioxidants, and avoid exposure to UV light. Additionally, they synthesize and accumulate compounds that can absorb UV radiation, acting like natural UV shields [133]. Different microalgal species exhibit varying levels of UV resistance, demonstrating the importance of these adaptations for survival in extreme environments. This ability to generate UV-shielding compounds has positioned microalgae as promising ingredients for natural sunscreen formulations [134,135].

Among these protective compounds, mycosporine-like amino acids (MAAs) are particularly effective against UV-induced damage. These small, water-soluble molecules possess a unique chemical structure, allowing them to absorb UV radiation and dissipate excess energy as heat, preventing ROS formation [136]. Over 30 types of MAAs have been identified, with examples including asterina-330, mycosporine-glycine, shinorine, porphyra-334, and palythine, each demonstrating strong UV absorption capabilities in various microalgal species. These molecules are critical for microalgal survival in high-stress environments, where they also increase resistance to extreme temperatures, salinity, and osmotic pressure. Additionally, MAAs work synergistically with other UV-protective compounds, such as scytonemin and pterins, enhancing overall photoprotection [134,137,138]. Beyond their UV absorption properties, MAAs also function as potent antioxidants, playing a role in activating the Nrf2 pathway, which regulates cellular defense mechanisms. Studies on human fibroblast cells indicate that MAAs can inhibit thymine-thymine dimer formation, effectively preventing UV-induced DNA mutations [139,140].

Due to their broad absorption spectrum (268–362 nm), MAAs effectively neutralize damaging free radicals, such as singlet oxygen and hydroxyl radicals, mitigating oxidative stress and protecting cellular integrity. For instance, mycosporine-glycine has been shown to specifically counteract ROS, reinforcing its protective potential [141]. These unique properties make MAAs ideal candidates for natural sunscreen formulations, offering both oxidative defense and UV protection, making them attractive ingredients for the cosmetic industry [140].

Beyond MAAs, other microalgal UV defense mechanisms contribute to photoprotection. Scytonemin and pterins, known for their broad-spectrum UV absorption, further enhance microalgae’s ability to withstand intense radiation exposure. Another specialized compound, sporopollenin, found in the cell walls of certain aeroterrestrial algae like *Scotiellopsis rubescens* and *Characium terrestre*, effectively blocks harmful UV-B rays while offering long-lasting protection. Its non-toxic and stable properties also allow it to bind heavy metals, contributing to additional environmental defense mechanisms [142]. In addition to producing UV-shielding compounds, microalgae actively protect skin from UV-induced damage by suppressing MMP production, which helps prevent ECM degradation. They also stimulate fibroblast proliferation, promoting skin regeneration and repair. Furthermore, microalgae coordinate defense mechanisms with other UV-protective molecules, reinforcing cellular resilience against environmental stressors [143,144].

Different microalgal species exhibit varied resistance to UV exposure, with certain species showing exceptional photoprotective capabilities. Research highlights Antarctic microalgae as particularly strong UV defenders, having evolved unique molecular adaptations to survive in harsh polar environments, where radiation exposure fluctuates due to ozone depletion. By leveraging these natural defense mechanisms, microalgae offer valuable insights into photoprotection, making them highly relevant for cosmetic and dermatological applications [[145], [146], [147]].

### 3.4 Hydration and moisture retention

Maintaining skin health requires supporting its natural functions, adaptability, and renewal processes, particularly in the outermost layers where hydration plays a crucial role. Younger skin retains more moisture, which contributes to firmness, resilience, and flexibility. However, with age, the ability to retain water diminishes, largely due to the decline of Natural Moisturizing Factors (NMFs) within the skin’s barrier, leading to dryness, rough texture, and reduced elasticity [148]. This progressive loss of hydration is exacerbated by hormonal changes, cellular aging, and environmental stressors such as sun exposure and genetic factors. A significant decrease in natural oil production, particularly in post-menopausal individuals, accelerates skin dehydration, requiring external moisturizing strategies to restore balance and maintain protection [128].

Moisturization is a fundamental aspect of skincare, as dryness can lead to discomfort, irritation, and compromised skin barrier function. When hydration levels decline, individuals may experience redness, white dry patches, cracking, and uneven texture, negatively impacting overall skin health and appearance [149]. Cosmetic formulations containing hydroxy acids (HA) have been employed in clinical practices to enhance skin hydration [150].

Beyond their well-documented bioactive properties, microalgae contribute to skin hydration and protection by strengthening the skin’s ability to retain moisture, ensuring long-term hydration. They also enhance ECM stability, reinforcing the skin’s structural integrity and supporting its protective functions. Additionally, microalgae play a crucial role in maintaining skin barrier integrity, helping to prevent excessive water loss and sustain overall skin health [[151], [152], [153], [154]]. Studies indicate that amino acids derived from *Spirulina* and *Chlorella* naturally improve water retention, minimize TEWL, and sustain cellular hydration levels [91].

Additionally, EPS from microalgae serve as powerful moisturizing agents, offering protection against enzymatic degradation while enhancing hydration. Their ability to retain moisture within the skin positions them as effective alternatives to hyaluronic acid in skincare formulations [155,156]. Certain polysaccharides from microalgae play a significant role in improving skin hydration and texture. For example, compounds such as chitinous polysaccharides, alginates, ulvans, laminarins, fucoidans, and agarose exhibit excellent hydrating effects, making them ideal for dry or aging skin when applied topically [157]. Among these, alginates and agar are widely incorporated into skincare formulations, such as moisturizing face masks, shaving foams, and lotions, where they form a protective layer on the skin, limiting moisture evaporation [151,158].

Furthermore, carrageenans (sulfated galactans) serve as thickening and gelling agents in body lotions, soaps, and shaving creams, improving product texture and hydration efficiency. Studies suggest that certain polysaccharides from brown algae offer superior moisturizing effects, expanding their potential in cosmetic formulations aimed at long-lasting hydration [151,158,159].

Beyond direct hydration, microalgae support skin barrier integrity, preventing moisture depletion due to environmental factors. Their mineral-rich polysaccharides exhibit hydrating and soothing effects, reinforcing skin defense mechanisms. Additionally, DNA extracts from various microalgae species demonstrate moisture-retention capabilities, further enhancing skin hydration and resilience [160]. Proteins and hydrolysates from microalgae, such as *Chlorella, Spirulina*, and *Porphyra*, have demonstrated a remarkable affinity for skin and hair, supporting moisture preservation and collagen integration. Their inclusion in moisturizers, hair care products, and dermatological formulations reinforces their role in cosmetic applications aimed at hydration and skin barrier repair [161].

By optimizing hydration mechanisms, reinforcing ECM function, and maintaining skin barrier stability, microalgae contribute to effective, long-term moisturization, making them valuable assets in dermatological and cosmetic formulations aimed at sustained hydration and skin health.

## 4 Scientific studies and clinical evidence

### 4.1 Overview of research on microalgae's impact on skin health

In recent years, the cosmetic industry has increasingly embraced natural and sustainable resources, with microalgae standing out due to their remarkable antioxidant, anti-inflammatory, and UV-protective properties [160,162]. These extracts are revolutionizing skincare by enhancing skin elasticity and firmness [163]. Several studies have explored the role of microalgae in enhancing skin health. Research indicates that *Chlorella* significantly boosts collagen production, helping to prevent wrinkles and improve skin firmness and smoothness [136,164,165]. Similarly, *Spirulina* has been found to promote collagen synthesis and reduce wrinkle depth [40,136], Additionally, specific extracts from *Planktochlorella nurekis* have shown promising potential in anti-aging treatments [166].

Microalgae also offer substantial anti-inflammatory benefits. These properties are particularly valuable in skincare formulations aiming to reduce redness and irritation. For instance, Badduri et al. [167] formulated an anti-acne topical treatment utilizing *Spirulina* extract, which is rich in phycocyanin protein. This study highlighted that antioxidant activity depends significantly on the concentration of phycocyanin. Furthermore, another investigation demonstrated that phycocyanin from *Spirulina* can notably reduce the production of inflammatory cytokines and enzymes, thereby alleviating skin inflammation and redness [84]. Additionally, strains like *Dunaliella salina, Chlorella sorokiniana*, and *Chlorella protothecoides* are recognized for producing valuable carotenoids, vital for skin care [[168], [169], [170]].

The exploration of microalgae for natural sun protection is also advancing [171]. Dr. Lotan [172] has patented sunscreen formulations incorporating microalgae, which can absorb sunlight and provide UV protection. Studies have shown that peptides from *Chlorella*, along with chlorophyll and carotenoids from *Chlorella vulgaris, Nostoc*, and *Spirulina* platensis, help protect the skin from UV damage [86,135]. *Nannochloropsis* has also demonstrated potential in shielding against UV-A and UV-B transmission [173]. Additionally, research has shown that certain microalgae can enhance the efficacy of sunscreens through the production of secondary metabolites with UV-absorbing properties [171]. Moreover, Mapoung et al. [174] conducted a study on the protective effects of *Spirulina* platensis extract on human skin fibroblasts exposed to UVB radiation. The extract, with an SPF value of 30.39, showed strong UVB absorption and antioxidant properties, reducing intracellular ROS without cytotoxic effects. It also inhibited tyrosinase activity and decreased secretion of inflammatory markers like TNFα., IL-6, and IL-8. These findings highlight the extract's photochemoprotective, antioxidant, and anti-inflammatory properties, making it a valuable ingredient in photoprotective cosmetic formulations. The phenolic and flavonoid compounds also provided UV protection, suggesting their potential as a bio-based UVB absorber with antioxidant effects.

Moreover, microalgae can enhance skin health through genetic modulation. Extracts from two microalgae species from the Blue Lagoon have been found to boost the expression of genes associated with skin health and protect against UVA radiation-induced damage [175]. Additionally, microalgae possess defense mechanisms against UV radiation, such as increasing levels of xanthophylls and MAAs, which help protect against UV-induced inflammation and oxidative stress [176]. For example, mycosporine-glycine from Chlamydomonas hedleyi effectively suppresses UV-induced COX-2 gene expression, indicating a link between COX-2 regulation and oxidative processes mediated by mycosporine-glycine [177].

Georgakis [178] performed an extensive evaluation of the cosmeceutical potential of aqueous extracts from *Trachydiscus minutus* microalgae, contrasting their properties with those of three well-known *Chlorella* strains: *Chlorella vulgaris, Chlorella. sorokiniana*, and *Chlorella minutissima*. The study revealed that *T. minutus* extracts exhibited superior antioxidant activity and notable elastase inhibition, making them promising candidates for anti-aging skincare products. Experiments on human skin cell cultures demonstrated that these extracts significantly reduced levels of inflammatory markers such as TNFα, IL-6, and IL-8. Additionally, they decreased UVA-induced ROS accumulation, thereby protecting the cells from oxidative damage. Similarly, Gunes et al. [179] explored the biomedical and pharmaceutical potential of skin creams enriched with bioactive *Spirulina* platensis extract. Their lab-based experiments demonstrated that *Spirulina* significantly promoted fibroblast cell proliferation and migration, essential for maintaining tissue integrity, managing inflammation, and facilitating scar formation. A skincare formulation with 1.125 % *Spirulina* extract notably boosted skin cell growth and enhanced the reactivity of collagen type 1, without showing genotoxic effects on human blood cells. These results highlight the promising role of *Spirulina* in skin regeneration and repair.

To better understand the effectiveness of bioactive compounds derived from microalgae, it is useful to compare them with conventional skincare ingredients. For example, hyaluronic acid is widely known for its exceptional moisture-retention capacity, enhancing skin hydration and elasticity. Similarly, sulfated polysaccharides from microalgae such as *Porphyridium* have demonstrated potent humectant properties, with clinical studies showing comparable improvements in skin moisture levels and barrier function. Additionally, clinical studies with carotenoid-rich microalgae like Haematococcus pluvialis reported improvements in skin moisture and barrier function comparable to those of conventional ingredients [180,181]. In terms of anti-aging effects, extracts from Spirulina and Chlorella have shown efficacy in reducing wrinkle depth and improving skin elasticity, effects that parallel those of retinol but without the associated risks of skin irritation or photosensitivity. These comparisons highlight microalgae as a sustainable and multifunctional alternative to traditional cosmeceutical ingredients, particularly in formulations targeting hydration, antioxidation, and skin repair [182,183].

Microalgae also offer considerable benefits for hair care. α-β carotene and vitamin E from *Pavlova nurekis, Chlorella vulgaris, Spirulina platensis*, and *Nannochloropsis oculata* play crucial roles in restoring hair vitality and scalp health [184]. *Schizochytrium limacinum* and *Chlamydomonas reinhardtii*, rich in polysaccharides, act as natural humectants, enhancing skin hydration by attracting environmental moisture. These algae also contain sterols beneficial for moisturizing creams and exhibit significant effects in biomedical treatments [96]. Secondary metabolites such as proteins, fatty acids, carotenoids, and pigments help prevent blemishes, restore damaged skin, and maintain moisture [159]. Their versatility is evident in both hair and skin care applications [96,185]. Jung et al. [186] evaluated six algae species—*Chaetoceros gracilis, Chlorella ellipsoidea, Nannochloropsis oculata, Pavlova lutheri, Phaeodactylum tricornutum*, and *Scenedesmus obliquus*—for their effectiveness in preventing hair loss and promoting scalp health in epidermal cells and hair follicle cells. Findings revealed that *Scenedesmus obliquus* and *Chaetoceros gracilis* were particularly effective.

### 4.2 Results from clinical trials and consumer experiences

Clinical research on cosmeceuticals derived from microalgae aims to evaluate the efficacy and safety of cosmetic formulations enriched with these extracts. These studies are designed to improve skin health by addressing issues like aging, hydration, and protection against environmental damage [187]. While existing research highlights the potential benefits of microalgae in skincare, more extensive trials are necessary to substantiate these findings and confirm the effectiveness of microalgae-based products [33,146,188]. To unlock the full potential of microalgae in skincare, it is imperative to delve into comprehensive studies that explore the intricate interactions between various microalgal species and human skin. Such research endeavors would illuminate the cellular pathways and molecular mechanisms through which these bioactive compounds exert their effects. This in-depth understanding is essential for validating the immediate efficacy of microalgae-based cosmetic products and uncovering their long-term benefits and potential applications in dermatology. Moreover, these findings could pave the way for innovative formulations that harness the unique properties of microalgae to address diverse skin concerns, ultimately elevating the standards of skincare and cosmetic science [189]. Table 4 outlines the clinical outcomes observed from evaluating cosmetic products formulated with various microalgal species, such as *Spirulina platensis, Scenedesmus rubescens, Neochloris oleoabundans, Chlorella vulgaris, Dunaliella salina*, and *Haematococcus pluvialis*.

Delsin et al. [190] explored the application of *Spirulina* in skincare products, developing a gel cream containing 0.1 % dry *Spirulina* extract. 40 healthy female participants applied this product twice daily, categorized into young (18–39 years) and mature (40–65 years) groups. Over 28 days, significant improvements were observed in skin hydration, reduction of sebum levels, dermal density, TEWL, and the distribution of keratinocytes. Similarly, Silva et al. [191] investigated the benefits of *Spirulina* in hair care, creating shampoos and conditioners containing *Spirulina* platensis and Ascophyllum nodosum extracts. Tested on 26 participants aged 18–35 with oily hair, the results after 28 days indicated a substantial reduction in combing force for both wet and dry hair, along with increased hair shine, demonstrating the effectiveness of these formulations on hair fibers.

The integration of microalgae in cosmetic formulations is revolutionizing skincare with its myriad benefits. Sulfated polysaccharides from *Porphyridium* have shown the ability to inhibit leukocyte migration to inflammation sites in vitro, with in vivo analyses demonstrating their effectiveness in reducing erythema in female volunteers aged 21–55 with skin inflammation [192]. Additionally, bioactive compounds such as C-phycocyanin from *Spirulina* platensis have exhibited strong antioxidant properties by inhibiting lipid peroxidation in rat liver models, as evidenced by clinical trial studies [193]. Furthermore, natural phycocyanin and its crosslinked nanoparticles, sourced from microalgae, have been effectively utilized in sunscreen formulations. Skin permeability tests in clinical trials suggest that phycocyanin is a promising alternative to synthetic sunscreen agents, addressing both environmental and health concerns [194]. Souza et al. [195] conducted a 3-month study on the photoprotective effects of *Spirulina* in sunscreen formulations with 44 participants. They found that combining *Spirulina* with UV filters enhanced the SPF value and provided better UVA protection in clinical trials compared to in vivo. Participants experienced improved skin health and elasticity, underscoring Spirulina's ability to regenerate the skin barrier and minimize water loss.

Campiche et al. [196] investigated the pigmentation effects of blue light (450 nm) on the skin using *Scenedesmus rubescens* extract. Conducted over 56 days with 33 female volunteers aged 21 to 41 with skin types III and IV, this randomized, double-blind, placebo-controlled study revealed that blue light exposure led to significant hyperpigmentation and skin reddening. Formulations containing 3 % *Scenedesmus rubescens* extract and 3 % niacinamide effectively mitigated these adverse effects, providing protective benefits against blue light-induced skin damage.

Putri et al. [197] examined the anti-aging properties of *Chlorella vulgaris* extract in a cream formulation. Tested on 50 participants aged 40–60, divided into groups using the *Chlorella* cream or a placebo, as well as on rabbits, the results indicated that the *Chlorella* cream significantly reduced wrinkle depth, enhanced skin firmness, and improved hydration, showcasing its anti-aging potential. Similarly, Havas et al. [198] assessed the effects of *Dunaliella salina* extract under intense solar exposure in a clinical trial with 25 women aged 35–60. Their study demonstrated that daily application of this carotenoid-rich extract significantly reduced advanced glycation end products (AGEs) and inflammatory markers like IL-6 and IL-8 while improving skin hydration and elasticity. Participants also observed fewer wrinkles and age spots, validating the extract's effectiveness against solar-induced aging. Conversely, Morocho-Jácome et al. [26] explored the anti-inflammatory properties of *Neochloris oleoabundans* extract in a hydrogel, tested on 20 healthy volunteers. While it did not affect skin hydration or TEWL, it showed notable anti-inflammatory effects by delaying erythema, as measured by laser Doppler.

Tominaga et al. [199] investigated the cosmetic benefits of astaxanthin derived from *Haematococcus pluvialis* in two human clinical trials. The first was an open-label study involving 30 healthy female subjects aged 20 to 60 years, and the second was a randomized, double-blind, placebo-controlled study with 36 healthy male subjects. Both studies reported significant improvements in skin wrinkles, age spot size, elasticity, skin texture, moisture content, and sebum oil levels, demonstrating the broad-spectrum efficacy of astaxanthin across various skin health parameters in both genders.

Jung et al. [200] and Choi et al. [201] examined the wound-healing potential of *Spirulina* incorporated into polycaprolactone (PCL) nanofibers. PCL, a biodegradable polymer, was used to enhance oxygen absorption and control water evaporation, critical factors for skin regeneration. Their studies indicated that *Spirulina*-PCL nanofibers significantly promoted wound healing and enhanced tissue repair by fortifying the skin’s antioxidant defenses against ROS in fibroblasts. However, the hydrophobic nature of PCL was noted to limit the moisturizing capacity of the nanofibers, suggesting an area for further refinement.

Despite the growing body of evidence supporting the potential benefits of microalgae in cosmetic formulations, it is important to acknowledge several methodological limitations that may affect the generalizability of current findings. Many of the available studies suffer from small sample sizes, short intervention durations, and limited demographic diversity. In addition, variations in the extraction methods, concentrations of bioactive compounds, and assessment protocols can lead to inconsistent results. Most importantly, long-term safety and efficacy data are lacking, as very few studies have examined the prolonged use of microalgae-based products in real-world settings. Therefore, there is a pressing need for large-scale, randomized, double-blind, placebo-controlled clinical trials with extended follow-up periods to robustly evaluate both the immediate and long-term effects of these promising bioactives on skin and hair health. Such studies would also help define optimal dosages and identify potential adverse reactions or sensitivities in diverse populations [1,202,203]. Table 3a

**Table 3a:** Exquisite insights into clinical advancements of microalgae-infused cosmeceuticals.

Microalgae	Participants	Formulation	Duration	Product	Outcomes	ref
*Spirulina platensis*	40 healthy females (18–39, 40–65)	0.1 % dry *Spirulina* gel cream	28 days	Skincare Gel Cream	Improved skin hydration, reduced TEWL, balanced sebum, increased dermal density, and better keratinocyte distribution.	[190]
*Spirulina platensis/Ascophyllum nodosum*	26 healthy individuals (18–35, oily hair)	Shampoos and conditioners	28 days	Hair Care Products	Reduced combing force (wet/dry hair), increased hair shine.	[191]
*Spirulina platensis*	44 participants (30–50 years)	Sunscreen formulations	3 months	Sunscreens	Enhanced SPF value, better UVA protection, improved skin health and elasticity, reduced water loss.	[195]
*Scenedesmus rubescens*	33 females (21–41, skin types III and IV)	3 % Scenedesmus rubescens extract, 3 % niacinamide	56 days	Anti-pigmentation Formulations	Mitigated blue light-induced hyperpigmentation and skin reddening.	[196]
*Chlorella Vulgaris*	50 participants (40–60), rabbits	Cream formulation	28 days	Anti-aging Cream	Reduced wrinkle depth, enhanced skin firmness, improved hydration.	[197]
*Dunaliella salina*	25 females (35–60)	Carotenoid-rich extract	56 days	Anti-aging Skincare	Reduced AGEs, decreased IL-6 and IL-8, improved skin hydration and elasticity, fewer wrinkles and age spots.	[198]
*Neochloris oleoabundans*	14 healthy participants (5 males, 9 females) (31.5 ± 12.6) (phototype II-IV skin)	Hydrogel formulation	2 h	Anti-inflammatory Gel	Delayed erythema, notable anti-inflammatory effects, no impact on skin hydration or TEWL.	[26]
*Haematococcus pluvialis*	30 females, 36 males (20–60)	Oral and topical astaxanthin	8 weeks (female) / 6 weeks (male)	Skin Health Supplements	Reduced wrinkles, smaller age spots, improved elasticity, better skin texture, increased moisture content, and balanced sebum oil levels.	[199]

## 5 Considerations and safety in using microalgae-based products

### 5.1 Potential allergies and reactions

Allergic reactions arise when the immune system mistakenly identifies a harmless substance as a threat, triggering an inflammatory response. While microalgae-based ingredients have gained recognition for their skin-enhancing properties, some individuals may experience sensitivities to certain proteins and polysaccharides present in these organisms. Studies suggest that structurally diverse proteins found in *Spirulina* and *Chlorella* may resemble environmental allergens, potentially leading to cross-reactivity [204,205]. For instance, phycocyanins from *Spirulina*, despite their antioxidant benefits, can act as sensitizing agents in certain cases. Similarly, complex carbohydrates in *Chlorella’s* cell wall may be perceived as foreign by the immune system, provoking an allergic response. Additionally, bioactive compounds such as phenolics, carotenoids, and peptides, prized for their role in skincare, can occasionally cause irritation or hypersensitivity. Although generally well-tolerated, their effect on the skin depends on individual susceptibility and formulation considerations [11,206].

The symptoms of allergic reactions to microalgae-based products are similar to those caused by other allergens. Dermatological issues, such as itchy rashes (contact dermatitis), are common skin reactions. Respiratory symptoms like including coughing, wheezing, or difficulty breathing, can occur, especially with powdered forms of microalgae, indicating an inhalation allergen. Gastrointestinal symptoms, such as nausea, vomiting, or diarrhea, may arise when microalgae products are ingested. In rare cases, exposure can lead to anaphylaxis, a severe and life-threatening reaction requiring immediate medical attention [31,206].

To prevent and manage these potential allergic reactions, several measures can be taken. Conducting a patch test by applying a small amount of the product to a discreet area of the skin can help identify any adverse reactions before widespread use. Ensuring that product labels list all ingredients allows consumers to avoid known allergens. Individuals with known allergies should consult healthcare professionals before using microalgae-based products. Additionally, developing hypoallergenic formulations can help cater to sensitive consumers and minimize allergenic potential [207].

Ensuring the safety of microalgae-based products involves rigorous testing and adherence to safety regulations. Clinical trials and dermatological assessments are essential to identify and mitigate potential allergens. Regulatory bodies such as the FDA and EMA provide guidelines for testing and labeling to ensure consumer safety. These regulations typically include allergenicity testing and the assessment of potential irritants. Through clinical trials, researchers can identify specific proteins or compounds responsible for allergic reactions and develop strategies to reduce their presence or impact. By understanding and addressing the potential allergenic risks, the cosmetics industry can better harness the benefits of microalgae while safeguarding consumer health. This balanced approach promotes the development of safe, effective, and sustainable cosmetic products that meet market demand and regulatory standards.

In addition to allergenicity concerns, the comprehensive safety of microalgae-based products necessitates rigorous toxicity testing and safety assessments before commercialization. These evaluations typically involve in vitro cytotoxicity assays, skin irritation tests, and phototoxicity studies to ensure that the bioactive compounds do not induce adverse cellular responses or sensitization under real-world usage conditions. Furthermore, repeated-dose dermal toxicity tests and mutagenicity assays (such as the Ames test) are conducted to identify any potential long-term or genotoxic effects associated with chronic exposure. The European Union's REACH regulation and the U.S. FDA guidelines require such safety assessments as part of the regulatory framework governing cosmetic ingredients. Additionally, microalgae extracts intended for leave-on formulations, such as creams and sunscreens, undergo human patch testing and, increasingly, advanced in vitro models to predict skin irritation and sensitization potential without relying on animal testing. These multidimensional safety evaluations ensure that microalgae-derived actives meet stringent safety standards, offering consumers not only efficacy but also confidence in the safety of these eco-friendly formulations [189,208].

### 5.2 Recommendations based on skin type and conditions

Microalgae-based skincare products provide a sustainable and adaptable approach to improving skin health. Their diverse bioactive components allow for targeted benefits tailored to specific skin conditions, optimizing effectiveness while minimizing adverse reactions [207].

For normal skin, microalgae offer essential nutrients that support vitality and help delay visible signs of aging. Their balanced composition ensures hydration, antioxidant protection, and cellular repair, maintaining a naturally radiant complexion. Dry skin benefits from deeply hydrating polysaccharides and nourishing essential EFAs, which replenish moisture and reinforce the skin barrier, reducing dryness and irritation. In oily and acne-prone skin, microalgae-derived anti-inflammatory and antimicrobial compounds help regulate sebum production and minimize breakouts, promoting balanced and clear skin. Sensitive skin finds comfort in hypoallergenic and soothing extracts such as *Dunaliella*, which reduce irritation and strengthen the skin’s tolerance to environmental stressors. Aging skin experiences rejuvenation through antioxidants, vitamins, and peptides that diminish wrinkles and improve elasticity, fostering a firmer and more youthful appearance. For hyperpigmentation, compounds like phycocyanin help even out complexion, while bioactive molecules such as carotenoids and vitamins work synergistically to regulate melanin production and enhance skin tone. Additionally, inflammatory skin conditions like eczema and psoriasis benefit from microalgae-based extracts that help reduce redness, itching, and discomfort while supporting long-term skin health [209,210].

Selecting formulations that align with individual skin needs enhances effectiveness and minimizes sensitivity risks. Patch testing and careful ingredient selection contribute to optimal results while ensuring a safe and tailored skincare experience. By harnessing the versatile properties of microalgae, skincare innovations can provide natural, effective solutions that support both dermatological health and environmental sustainability.

### 5.3 Proper usage and dosage guidelines

Ensuring the proper usage and dosage of microalgae-based cosmetic products is vital for their effectiveness and safety. By following these guidelines, consumers can achieve optimal results and minimize the risk of adverse reactions. A thorough understanding of the product label and the manufacturer's instructions is essential, as these details provide crucial information on recommended usage, appropriate dosage, and necessary precautions.

The effectiveness of microalgae-based formulations depends significantly on the concentration of bioactive compounds they contain. Factors such as the species of microalgae, extraction methods, intended benefits, and product type play a crucial role in determining these concentrations. Typically, these formulations are rich in carotenoids, polyunsaturated fatty acids (PUFAs), polysaccharides, proteins, and peptides, ensuring both safety and efficacy [211].

For instance, Fucoxanthin, a carotenoid found in certain microalgae strains, is typically used at concentrations of 0.02–0.1 % in cosmetic formulations for its antioxidant and anti-aging properties. Beta-glucan from microalgae, known for its skin-soothing and moisturizing effects, is usually included in concentrations ranging from 0.5 % to 3 % in skincare products. EPA is often used at 0.05–0.2 % concentrations for its anti-inflammatory benefits [212]. MAAs, valued for their UV-absorbing capabilities, are utilized within the 0.01–0.05 % range in sunscreens [143]. Additionally, Astaxanthin, a potent antioxidant, is typically included at 0.01–0.05 % for UV protection, while phycocyanin, a natural photoprotective agent, is used at concentrations ranging from 100 to 1000 μg/mL [213]. Microalgal polysaccharides and peptides serve as effective moisturizers and anti-wrinkle agents at concentrations ranging from 0.5 % to 5 %. Whitening agents derived from microalgae, prized for their skin-brightening properties, are generally used at concentrations between 0.1 % and 1 %. Furthermore, microalgal extracts can enhance the texture of cosmetic products, with their concentration varying depending on the specific extract and desired effect. These extracts often include compounds with skin-brightening properties, used at low concentrations, typically between 0.1 % and 1 % [143]. Phycocyanin, acting as a natural photoprotective agent, is commonly included at concentrations ranging from 100 to 1000 μg/mL in sunscreen formulations [214]. Manufacturers rigorously assess stability and safety to determine optimal concentrations of bioactive compounds in their formulations. These evaluations ensure efficacy and user safety. Therefore, consumers must check product labels for ingredient concentrations. This attention to detail guarantees that products deliver benefits such as moisturizing, anti-aging, and UV protection without adverse effects [213,214].

By adhering to these guidelines and understanding the specific roles and concentrations of various bioactive compounds, users can effectively and safely incorporate microalgae-based products into their skincare routine, maximizing benefits and minimizing potential side effects.

## 6 Future prospects and innovations in microalgae skincare

### 6.1 Development of new products

The development of new cosmetic products utilizing microalgae is an elaborate and complex process that capitalizes on the unique biochemical properties of these microorganisms. Initially, extensive research is undertaken to identify and screen a variety of microalgae species for their beneficial attributes. This involves laboratory experiments aimed at isolating bioactive compounds such as antioxidants, anti-inflammatories, and moisturizers. Many microalgae species naturally synthesize recombinant proteins and peptides with distinct sequences and configurations, which are challenging to replicate through conventional synthetic methods. Additionally, secondary metabolites produced by microalgae, including phenolics, flavonoids, and alkaloids, have shown significant potential in skincare applications due to their potent biological activities. These compounds can be incorporated into anti-aging creams and serums to reduce oxidative stress and improve skin elasticity. Researchers could explore the potential of alkaloids in treating hyperpigmentation and skin discoloration, formulating products that specifically target melanin production and distribution. Upon identification, advanced extraction methods, including solvent extraction and supercritical CO_2_ extraction, are employed to obtain these compounds in their purest form [[215], [216], [217]].

The importance of using microalgae in cosmetic and skincare products lies in their natural and sustainable origin, their ability to produce a wide range of bioactive compounds, and their minimal environmental impact. Microalgae are a renewable resource that can be cultivated using non-arable land and non-potable water, making them an eco-friendly alternative to traditional ingredients. Their bioactive compounds, such as antioxidants, anti-inflammatories, and UV-protective agents, offer effective skincare benefits while reducing reliance on synthetic chemicals. Moreover, the cultivation and processing of microalgae generate fewer greenhouse gases and require less energy compared to conventional agricultural practices, contributing to a lower carbon footprint [[6], [7], [8], [9], [10]]. Consumer demand for natural and organic products is driving significant growth in the cosmetics market, expected to reach $295.65 million by 2029. Continuous research and development are expanding the use of microalgae in beauty products [218]. Consumers favor minimalist formulations, natural ingredients, and sustainable practices. Transparency in sourcing and production builds consumer trust [219]. Microalgae-based cosmetics align with these consumer preferences, making them ideal for skincare products.

These bioactive compounds are then meticulously integrated into cosmetic formulations. The formulation process requires the selection of complementary ingredients that not only amplify the efficacy of the bioactive compounds but also ensure the product's overall stability and longevity. Each ingredient's concentration must be meticulously optimized, and the formulation undergoes rigorous testing to confirm its efficacy and safety [220,221]. This phase includes an array of tests such as stability testing, compatibility testing, and performance evaluation under various environmental conditions to ensure the product's robustness [222]. Ensuring that these cosmetic products are skin-compatible and have minimal side effects is essential. This involves selecting ingredients that are gentle on the skin and conducting thorough dermatological tests to identify potential irritants or allergens. Formulating products with natural and non-toxic components can help minimize adverse reactions and enhance consumer safety and satisfaction [223]. Developing multifunctional skincare products that leverage the diverse biological activities of secondary metabolites can address multiple skin concerns simultaneously. For example, a moisturizer enriched with phenolics, flavonoids, and peptides can provide hydration, antioxidant protection, and anti-inflammatory benefits in one application [224].

Future research should focus on high-throughput screening techniques to rapidly identify and characterize novel bioactive compounds from an extensive library of microalgae species. This approach can significantly accelerate the discovery process and ensure that the most promising candidates are selected for further development [225]. Advanced metabolic and genetic engineering strategies can be employed to enhance the production of specific bioactive compounds [226,227]. By manipulating metabolic pathways, researchers can increase the yield of desired compounds, reduce the production of unwanted by-products, and optimize the overall productivity of microalgae cultures [228]. Moreover, the use of secondary metabolites as natural preservatives in cosmetic formulations is another promising area. Phenolics and flavonoids possess antimicrobial properties that can help extend the shelf life of products without the need for synthetic preservatives, aligning with the consumer demand for natural ingredients [124].

Integrating nanotechnology into the development of microalgae-based cosmetic products can further enhance their efficacy. Nanoparticles can be used as carriers for bioactive compounds, improving their stability, bioavailability, and targeted delivery to specific skin layers. This approach can also facilitate the controlled release of active ingredients, ensuring sustained benefits over extended periods [229]. Developing synergistic formulations that combine multiple bioactive compounds from different microalgae species can provide enhanced skin benefits. By leveraging the complementary effects of various compounds, these formulations can offer comprehensive solutions for complex skin conditions, such as aging, hyperpigmentation, and inflammation.

Many existing studies have primarily focused on isolated species of microalgae and their individual bioactive compounds, often neglecting the potential synergistic effects of combining m*ultiple species*. Furthermore, there is limited research on the use of secondary metabolites from microalgae as natural preservatives and their application in multifunctional skincare products. Our research addresses these gaps by conducting a comprehensive analysis of various microalgae species and exploring innovative applications of secondary metabolites.

The formulations are subjected to extensive clinical trials to ascertain their safety and efficacy. Dermatological tests are conducted to evaluate potential irritation or allergic reactions, while efficacy studies are performed to measure the impact on skin health. Regulatory approvals from authoritative bodies such as the FDA or EMA are sought to ensure compliance with safety standards [37,230,231]. Following regulatory approval, the production process is scaled up from laboratory to industrial levels, maintaining stringent quality control measures to ensure consistency and reliability of the final product. Sustainable packaging solutions, including the use of biodegradable and recyclable materials, are implemented to minimize the environmental footprint [232,233].

Moreover, the advent of bioprinting technology opens up new possibilities for personalized skincare solutions [234]. By utilizing bioinks derived from microalgae, customized skincare products can be printed to match individual skin profiles, ensuring optimal efficacy and minimizing the risk of adverse reactions. Innovations in smart packaging can provide additional value to microalgae-based cosmetic products. Packaging materials embedded with sensors can monitor product freshness, detect contamination, and provide real-time information to consumers about the product's condition. This technology can enhance consumer trust and satisfaction [235].

Implementing comprehensive sustainability metrics to assess the environmental impact of microalgae cultivation, extraction processes, and product formulations is crucial. These metrics can guide the optimization of production methods, reduce resource consumption, and ensure that the overall environmental footprint of microalgae-based products is minimized [236,237]. While cutting-edge technologies such as 3D bioprinting and synthetic biology hold long-term promise, near-term innovations are expected to drive the practical integration of microalgae into mainstream skincare. These include advanced extraction techniques, AI-enabled formulation development, and the expansion of sustainable cultivation systems like photobioreactors, which together can enhance scalability, affordability, and efficacy of microalgae-based products [238,239].

Artificial Intelligence (AI) can play a pivotal role in the future development of microalgae-based cosmetic products. AI can be utilized to analyze large datasets from clinical trials, consumer feedback, and market trends to optimize product formulations and predict emerging trends. AI-driven algorithms can identify the most effective combinations of bioactive compounds and their optimal concentrations, enhancing the efficacy and safety of the products. Moreover, AI can enable the personalization of skincare products by analyzing individual skin profiles and recommending formulations tailored to specific skin types and concerns. AI can also facilitate the automation of quality control processes, ensuring consistent product quality and reducing the risk of human error. Additionally, AI-powered simulations can accelerate the discovery of new bioactive compounds by modeling the interactions between microalgae compounds and skin cells at a molecular level, providing deeper insights into their mechanisms of action [240,241].

Finally, comprehensive marketing and distribution strategies are devised to effectively introduce the product to the market. Emphasizing the natural, sustainable, and efficacious attributes of microalgae-derived products can attract environmentally conscious consumers. Utilizing green certifications and transparent communication about the product’s benefits can enhance consumer trust and drive market adoption [242,243].

Our approach is distinguished by its comprehensive analysis of various microalgae species, in contrast to many studies that focus solely on single species. We investigate the unique bioactive compounds and their synergistic effects, thereby enhancing the diversity and effectiveness of our skincare formulations. Additionally, our study places significant emphasis on economic and sustainability aspects through thorough cost-benefit analyses and sustainable production processes, ensuring that our solutions are both effective and environmentally responsible. They meet global trends for clean beauty products that are free from GMOs, animal-derived ingredients, and animal testing. Furthermore, the demand for multifunctional formulations highlights microalgae as a key ingredient in skincare products [244].

By embracing these innovative approaches and continuously advancing our scientific understanding of microalgae, we can unlock the full potential of these microorganisms and create cutting-edge cosmetic products that meet the evolving needs of consumers, are gentle and compatible with the skin, have minimal side effects, and contribute to a more sustainable and environmentally conscious beauty industry. These innovative proposals underscore the potential of secondary metabolites in advancing the efficacy and sustainability of microalgae-based cosmetic products. By exploring these novel applications, we can continue to push the boundaries of skincare science and meet the growing consumer demand for effective and natural skincare solutions.

### 6.2 Research opportunities and novel applications

Research opportunities and novel applications of microalgae in the cosmetics industry represent a dynamic and expanding field. The unique properties of microalgae, combined with biotechnological advancements, offer numerous possibilities for innovative research and development. One key area is identifying and characterizing new microalgae species with bioactive compounds such as antioxidants, anti-inflammatory agents, and UV-protective molecules, which provide natural and effective alternatives to synthetic ingredients [245,246].

Optimizing microalgae cultivation techniques is another promising research avenue. Enhancing growth conditions like light intensity, nutrient availability, and CO_2_ concentration can improve bioactive compound yields. Advanced systems like photobioreactors and closed-loop systems support more efficient and sustainable production [247,248].

Utilizing artificial intelligence to develop models that predict optimal cultivation conditions for different microalgae species is a promising research direction. These models can analyze variables such as light, temperature, and nutrient levels to maximize the yield of bioactive compounds. AI-driven models can analyze large datasets and identify patterns that optimize cultivation conditions, leading to more efficient and cost-effective processes [249,250].

Genetic engineering also presents significant opportunities. Through genetic modification, scientists can boost the production of specific bioactive compounds or introduce pathways for new compounds with desired cosmetic properties, improving microalgae strains' resilience and productivity [247,248,251]. For example, employing CRISPR-Cas9 technology to precisely edit the genomes of microalgae can enhance the production of specific bioactive compounds and introduce new metabolic pathways for novel compounds with superior cosmetic properties. This technology allows for precise modifications of the microalgae genome, significantly improving the efficiency and effectiveness of microalgae-based cosmetic products [252].

Investigating the interactions between microalgae extracts and the skin microbiome can lead to the development of products that benefit the skin directly and promote a healthy and balanced skin microbiome. The skin microbiome plays a crucial role in skin health, and understanding these interactions can lead to improved skin health and reduced risk of skin conditions such as acne and eczema [253]. Beyond skincare, microalgae-derived pigments and dyes offer sustainable alternatives to synthetic colorants in makeup and hair care products. These natural pigments are biodegradable and non-toxic, aligning with the growing demand for eco-friendly products [254]. Microalgae-based biopolymers and biofilms are explored for biodegradable packaging materials, reducing environmental impact [255].

Developing and implementing green extraction techniques such as supercritical CO_2_ extraction and enzyme-assisted extraction to obtain bioactive compounds from microalgae in an environmentally friendly manner can reduce the use of harmful solvents. Sustainable extraction methods use environmentally friendly techniques, minimizing the environmental impact and improving the sustainability and safety of microalgae-based cosmetic products [[256], [257], [258]].

Innovative delivery systems, such as liposomes and nanocarriers, enhance the stability and bioavailability of microalgae-derived compounds in cosmetic formulations, ensuring effective delivery of active ingredients to the skin [50]. Exploring the use of 3D bioprinting technology to create personalized skincare products that incorporate microalgae-derived compounds, tailored to individual skin types and conditions, is another innovative direction. 3D bioprinting allows for the precise fabrication of personalized skincare products, leading to more effective and targeted skincare solutions [234]. Integrating microalgae into holistic skincare regimens involves using supplements rich in vitamins, minerals, and EFAs to promote skin health from within, complementing topical treatments [259].

Overall, the research opportunities and applications of microalgae in cosmetics are vast. Continued investment will likely lead to groundbreaking discoveries and innovations, transforming skincare and cosmetic products. Integrating sustainable practices and advanced biotechnological techniques will ensure microalgae remains at the forefront of cosmetic research and development. Developing microalgae-based products specifically designed to protect the skin from urban pollution can incorporate compounds that form a barrier against pollutants and detoxify the skin, reducing the risk of skin damage and improving overall skin health. By pursuing these innovative research proposals, the cosmetics industry can harness the full potential of microalgae, leading to the development of next-generation skincare and cosmetic products that are effective, sustainable, and tailored to meet the needs of modern consumers.

### 6.3 Environmental impact and sustainability in production

Microalgae present an environmentally conscious solution for the production of cosmetic products. Thriving in non-arable lands and diverse water sources, microalgae help conserve arable land and freshwater resources. Advanced water recycling techniques, alongside the implementation of closed-loop systems, ensure that water used in microalgae cultivation is continuously recycled and purified, minimizing the overall water footprint of the production process. These measures reduce water consumption and treat pollutants, while the utilization of renewable energy sources such as solar and wind power lowers the carbon footprint [[260], [261], [262]].

Microalgae possess the unique ability to absorb and utilize industrial CO_2_ emissions for growth, providing a solution to combat climate change. By integrating microalgae cultivation with industrial processes, companies can effectively reduce their carbon footprint while producing valuable bioactive compounds for cosmetic applications. Nutrient management and the utilization of industrial CO_2_ for growth further reduce environmental impact [263]. Strict containment protocols are implemented to prevent the release of non-native species into the environment, preserving biodiversity and maintaining ecological balance [264].Embracing a circular economy, microalgae by-products are repurposed into biofertilizers, animal feed, and bioenergy, maximizing resource efficiency and minimizing waste [265]. Designing local microgrids powered by energy produced from microalgae can enhance sustainability and energy self-sufficiency for communities [266]. An innovative approach involves the development of bio-based sensors from microalgae to monitor environmental quality in real-time. These sensors can quickly detect changes in environmental conditions, aiding in faster and more accurate management decisions [267]. Creating vertical farming systems for microalgae, which require less space and offer higher productivity, can significantly enhance the scalability of microalgae production. These systems can be utilized in urban areas or confined spaces, increasing overall yield [268]. Using blockchain technology to increase transparency in the microalgae supply chain can assure consumers that their products are sourced sustainably and produced in compliance with environmental standards [269].

## 7 Conclusion

In conclusion, microalgae have revolutionized the cosmetics industry with their diverse bioactive compounds, including antioxidants, vitamins, minerals, essential fatty acids, polysaccharides, peptides, and amino acids. These compounds offer significant skin benefits such as reducing wrinkles, improving skin elasticity and firmness, fading dark spots, and accelerating wound healing. The integration of microalgae in cosmetic formulations provides a unique approach to skincare, utilizing natural ingredients that are both effective and eco-friendly. However, comprehensive clinical trials and standardized guidelines are necessary to ensure the efficacy and safety of microalgae-based products, requiring rigorous scientific validation and regulatory oversight

Innovations such as bio-based sensors, vertical farming systems, local microgrids powered by microalgae, artificial intelligence, and blockchain technology can significantly advance the cosmetics industry by optimizing cultivation, extraction, and production processes. These technologies enhance efficiency and sustainability, contributing to high-quality, eco-friendly products. Comprehensive analyses of various microalgae species, research on specific bioactive mechanisms, and the development of sustainable production and biodegradable packaging will further enhance the economic viability and environmental sustainability of microalgae-based products. Leveraging AI for data analysis, product personalization, and production optimization can revolutionize the industry, ensuring personalized skincare solutions tailored to individual skin profiles. Continued investment in research and sustainable practices will address these challenges, fostering a more innovative and sustainable future for the cosmetics industry. Furthermore, the innovations discussed in this paper highlight novel applications of microalgae, focusing on integrating advanced technologies and sustainable practices to develop highly effective and eco-friendly beauty solutions. These advancements underscore the transformative potential of microalgae, paving the way for a new era in cosmetics. Embracing these advancements can meet the growing consumer demand for natural and sustainable products while contributing to broader environmental goals such as reducing carbon emissions, conserving water, and minimizing waste. The transition towards a circular economy, where microalgae by-products are repurposed into biofertilizers, animal feed, and bioenergy, maximizes resource efficiency and minimizes waste.

To fully harness the transformative potential of microalgae in cosmetics, it is essential to foster stronger collaboration between academia and industry. Such partnerships can accelerate the translation of scientific discoveries into market-ready products, ensure rigorous safety and efficacy validation, and drive innovation in eco-friendly, high-performance formulations. By working together, researchers, manufacturers, and regulatory bodies can create a thriving ecosystem that not only delivers cutting-edge skincare solutions but also contributes to global sustainability goals.

In summary, further research and informed usage are crucial to fully realize the potential of microalgae and ensure safety and efficacy in their applications. By harnessing the unique properties of microalgae, the cosmetics industry can pave the way for a future that is not only technologically advanced but also environmentally conscious and consumer-friendly. This comprehensive approach will enhance the quality of cosmetic products, align the industry with global sustainability objectives, and ultimately benefit both consumers and the planet.

## CRediT authorship contribution statement

**Negin Chinjoo:** Writing – review & editing, Writing – original draft, Methodology, Data curation, Conceptualization. **Abooali Golzary:** Writing – review & editing, Validation, Project administration, Methodology, Data curation, Conceptualization.

## Declaration of competing interest

The authors declare that they have no known competing financial interests or personal relationships that could have appeared to influence the work reported in this paper.

## Data Availability

The data that has been used is confidential.
